# Two-year impact of an educational intervention in primary care on blood glucose control and diabetes knowledge among patients with type 2 diabetes mellitus: a study in rural China

**DOI:** 10.1080/16549716.2021.1893502

**Published:** 2021-04-07

**Authors:** Shaofan Chen, Dongfu Qian, Bo Burström

**Affiliations:** a Health Outcomes and Economic Evaluation Research Group, Stockholm Centre for Healthcare Ethics, Department of Learning, Informatics, Management and Ethics, Karolinska Institutet, Stockholm, Sweden; b Equity and Health Policy Research Group, Department of Global Public Health, Karolinska Institutet, Stockholm, Sweden; c School of Health Policy and Management, Nanjing Medical University, Nanjing, China; d Center for Health Policy Studies, Nanjing Medical University, Nanjing, China; e Center for Global Health, Nanjing Medical University, Nanjing, China

**Keywords:** Diabetes care, educational intervention, primary care, rural China, long-term impact

## Abstract

**Background**: Type 2 diabetes mellitus is increasing in rural China and should be managed in primary health care, but knowledge is lacking. Educational interventions have been implemented but not followed up long-term.

**Objective**: The study aimed to assess the long-term impact of an educational intervention on patients’ diabetes knowledge and fasting blood glucose (FBG) level, and whether these outcomes differed between two rural counties.

**Methods**: The study was nested in an educational intervention project in primary health care in Jiangsu province. Patients with type 2 diabetes mellitus from Huaiyin county and Gaochun county were randomly divided into an intervention group receiving an educational intervention and follow-up visits, and a control group with standard care. Questionnaires and medical records, including FBG level and diabetes knowledge score, were compared, at baseline in 2015 and two follow-ups, in 2016, and 2017, respectively. A paired t-test and two mixed-effects linear regression models were used.

**Results**: The diabetes knowledge score increased in the intervention group in 2016 and in 2017, compared with 2015. The FBG level decreased in 2016 compared with 2015 in the intervention and control groups. Comparing data in 2015 and 2017, there was no significant change in FBG level in the intervention or control group, but the diabetes knowledge score increased in the intervention group both in 2016 and 2017. A significant association between FBG level and the interaction of time and group, suggesting a long-term effect, was only found in Gaochun county in 2017.

**Conclusion**: The educational intervention improved the diabetes knowledge score in the intervention group, while no significant improvement was found in the control group in both year 2016 and 2017. Meanwhile, the intervention had a positive impact on FBG level in the intervention group in 2017. Patients in Gaochun county had better improvement in both diabetes knowledge and controlling FBG level, compared with Huaiyin county.

## Background

Type 2 diabetes mellitus (T2DM) remains a major issue to people’s health globally, as the number of patients has almost quadrupled in the last 30 years [1]. It is becoming a threat to people’s health in China as well. The overall prevalence of T2DM in China increased dramatically from 0.7% in 1980 to 12.8% in 2017 [2,3]. The fast urbanization, population aging, and lifestyle changes, with increasing obesity, may account for the rapid growth of T2DM [4]. Rural China is experiencing an even more difficult situation regarding T2DM, with a faster increasing rate of incidence than in urban areas, while the awareness, treatment, and control of diabetes remain lower [5].

There are three main types of health insurance for Chinese residents: the Urban Employee Basic Medical Insurance (UEBMI), Urban Residence Basic Medical Insurance (URBMI), and the New Rural Cooperative Medical Scheme (NRCMS) [5]. Patients will pay their consultation or medication fee in the first place, and get reimbursements afterwards. Primary health care (PHC), which has been proven to be the critical setting for the effective management of chronic diseases, is still facing challenges in rural China [6–8]. The PHC institutions in rural China consist of publicly owned township health centres and village clinics, where physicians and nurses often have low levels of training [7,8]. Chronic disease care in rural China is dominated by costly hospital care, and the integration between hospitals and PHC is lacking [8]. The Chinese health care reform launched in 2009, aimed to provide opportunities for PHC development for chronic disease care in rural China [8]. As one of the most essential components of the reform, PHC was strengthened and the subsidies were increased by the central government [7]. The accessibility and affordability of primary were suggested to be improved by shifting care from hospitals to PHC [9].

Hence, efforts are needed for strengthening and increasing knowledge in PHC to improve the care of patients with chronic diseases. Two systematic reviews found a number of educational interventions for T2DM patients implemented in China and other parts of the world, with the aim to improve glucose control and empower patients themselves better to manage their disease [10,11]. Family and community based educational interventions have been widely employed, and the reviews concluded that educational interventions could be of benefit to patients by improving blood glucose level and diabetes knowledge [10,11].

However the evidence supporting the efficacy of educational interventions is limited to short-term studies of typically under one year [10,11]. Few studies with a long-term follow-up were found in different settings [12–15]. We only found two Chinese language studies focused on urban China with an educational intervention longer than one year [16,17]. No study was found with such long-term intervention in rural China.

A project ‘Studying the Vertical Integration Strategy of Chronic Disease Service Based on Multiple Incentive Mechanism in Rural China’ has been implemented from 2015 to 2017, to optimize the care of patients with T2DM and primary hypertension in three pilot counties in rural areas of Jiangsu Province [18]. The project aimed to shift the care of T2DM and primary hypertension from hospital to PHC, through implementing an educational intervention for patients and physicians and nurses in PHC [18]. Two studies from this project have reported that the intervention had a positive impact on improving patients’ diabetes knowledge, health-related quality of life, and reducing fasting blood glucose level at one-year follow-up [19,20]. The present study is a part of this project and focuses on the 2-year follow-up of the educational intervention, and its impact on (a) patients’ diabetes knowledge; (b) the fasting blood glucose (FBG) level; and (c) whether these outcomes differed in different rural counties. More details of the intervention are presented in a study protocol [18].

## Methods

### Study setting

The study was conducted in Jiangsu Province, located in south-east China. In 2018, Jiangsu Province had a population of 80.5 million, of which 24.5 million residents lived in 87 rural counties [21]. Jiangsu Province is one of the most developed provinces with the second largest gross domestic product in China [21]. It can be divided into three parts by geographic features: the north part (less developed, 37 counties), the middle part (average developed, 11 counties), and the south part (most developed, 39 counties) [22]. In the present study, cluster randomization was used when selecting the counties and townships. One county from each of the three parts was randomly selected by the research team as a study area.

The research team in Nanjing Medical University (NMU) was responsible for selecting counties for the study. Huaiyin, Jingjiang, and Gaochun were selected in the north, middle and south part, respectively. Huaiyin consists of 14 townships, while Jingjiang and Gaochun both have eight townships, respectively [22]. In each county, 2 to 4 townships were randomly selected as the intervention area, by the local county-level Health and Family Planning Commissions (HFPC). HFPC is the health authority which is responsible for raising health awareness and education, family planning, ensuring the accessibility of health services, monitoring the quality of health services at the local level [9]. According to the sociodemographic features, the economic development situation, and the medical care level, the other 2 to 4 comparable control townships were subsequently selected in the same county. In total, 9 intervention townships, and 9 control townships were part of the study.

### Study population

Participants were selected in the respective counties and townships in 2015. The doctors in township health centres contacted patients with T2DM who met the inclusion criteria according to their records. Patients were asked to participate in the project, and written informed consent was obtained.

The inclusion criteria were: met the diagnostic criteria of the Chinese Guidelines on the Prevention and Treatment of Type 2 Diabetes Mellitus [4]; were aged 35–75 years; had lived in the county for more than two years with no records of moving within the last year; had personal records in the chronic disease management information system in the township health centres; had accepted the chronic disease service provided by the PHC institutions; and were willing to participate in the project and also be willing to acquiring new knowledge about diabetes. Patients were excluded from the study if they had severe diabetes complications, or they were diagnosed with any other severe disease, or they were pregnant or had psychiatric disorders.

### The intervention

The intervention aimed to strengthen collaboration between hospital care and PHC, to improve patients’ diabetes knowledge and improved fasting blood glucose (FBG) level, and to improve the knowledge and management of diabetes among health care professionals in PHC. The intervention for patients included education lectures, follow-up services, and special medical services, while health care professionals in PHC received professional skills training, team discussions, and regular meetings.

The educational intervention was conducted by service teams, assembled by the county-level HFPC in the intervention areas. Each service team consisted of physicians, nurses, public health physicians, and diabetes specialists, from all three-level health care institutions in rural China (county-level hospitals, township health centres, and village clinics).

The intervention for patients with T2DM lasted for 2 years, from 2015 to 2017. Apart from the routine service, patient participants in the intervention areas received services including: 1) health education lectures every two months; 2) periodical follow-up go-to-door visits with an annual physical examination; 3) special medical services (including helping patients with medical treatment, transfer treatment, return visit, and clinical care). The county level HFPCs were responsible for the quality control process. In the first year, the research team acted as the advisor for the implementation, while the local HFPC and service team took full responsibility for implementing the intervention in the second year. Jingjiang county discontinued the intervention in 2016 for administrative reasons.

The health education lectures contained information on: 1) basic information on diabetes, including typical symptoms, the basic diagnose criteria, diabetes-related complications, and basic epidemiological facts; 2) self-management strategies, including instructions for monitoring blood glucose at home, food recommendations, and suggestions about how to use medication; 3) advice on physical exercise and diet therapy; 4) advice to patients when having high blood glucose level, such as balancing sugar, protein, and fat intake; quitting smoking and drinking; recommending bean products; and controlling cholesterol intake; 5) introduction of measuring blood glucose and severe acute complications; 6) prevention of diabetes, based on the Triple Prevention Strategy of Diabetes, which is recommended in the guidelines for T2DM [4]. The detailed content of the lectures was decided by the service team in each county. The diabetes experts in the service team gave the lectures to the patients in the intervention areas.

Periodical follow-up home visits were conducted every two months. Doctors in township health centres in the intervention areas paid a home visit and measured blood glucose level among patients. The patients received counseling from doctors according to their blood glucose level and eating or physical exercise records. The doctors also listened to patients’ descriptions of their feelings about the education lecture. The intervention is explained more in detail in the study protocol [18].

Patients in the control areas received routine healthcare services as usual, including clinic visits to a physician and referrals as required according to the patient’s condition, and FBG test every four to six months, and were provided diabetes knowledge leaflets once a year [4]. Detailed information about the intervention for patients is listed in Appendix Table A1.

The intervention for health care professionals in PHC included: 1) professional skills training from the county-level hospitals; 2) team discussions regarding patient cases; 3) regular meetings to discuss teamwork progress; 4) technical checks to inspect prevention and treatment plans; 5) performance appraisals, which were controlled by county level HFPCs, to encourage professionals to participate actively in the study. A previous study focused on effects of this intervention among health care professionals in PHC, and found a positive impact on their professional diabetes skills, knowledge, attitudes, practices, and types of services they were able to provide at a one-year follow-up [23].

### Outcomes

The research team designed and pilot tested a questionnaire with 77 questions, to ask the patients during the baseline data collection in October 2015, at the first follow-up data collection in October 2016, and at the second follow-up data collection in July 2017. The questionnaire concerned 10 parts related to patients’ perspectives about diabetes and diabetes care, including diabetes knowledge and sociodemographic characteristics. The patients’ sociodemographic characteristics included age (in years); sex (male vs. female); marital status (married/cohabiting vs. single); the level of education (lower vs. higher); occupation type (farmer/housework vs. other); and the duration (in years) of being diagnosed with T2DM. Participants with primary school or less were classified as having low education; those with higher than primary school (middle school/high school/junior college/bachelor or higher) were classified as having high education. The FBG level, measured in mmol/l from a venous blood sample, was also registered during data collection. For the present study, we used data collected at baseline, and two follow-ups in Huaiyin (north) and Gaochun (south) county.

We analysed the FBG level, diabetes knowledge score and sociodemographic characteristics among patients, in 2015, 2016, and 2017. The diabetes knowledge score was measured as a summary score, based on the correct response to nine questions in the questionnaire. The specific nine questions related to diabetes knowledge are listed in Appendix Table B1.

### Data analysis

The independent t-test and Pearson’s χ2-test was used to study differences in sociodemographic characteristics between the intervention and control group. In order to analyse the changes between the baseline and the two follow-up data collection points within the intervention group and the control group, a paired t-test was used to compare the mean difference in FBG and diabetes knowledge score. Two mixed-effects linear regression models were used, one was to investigate the associations between the diabetes knowledge score and the interaction of time and group. The other model was to investigate the associations between the FBG level and the interaction of time and group. Participants were assumed to be random effects, and participants’ identification number was introduced into the models as a random intercept to account for the possible clustering. Time, group, and the interaction of time and group were treated as fixed effect. The participants’ sociodemographic characteristics including age, years having been diagnosed with T2DM, sex, educational group, occupation group, and marital status were introduced into both the models as covariates. SPSS version 22.0 [24] and Stata 11.0 [25] were used to analyse the data. All statistical tests were carried out at a 5% significance level.

## Results

**Figure 1.**
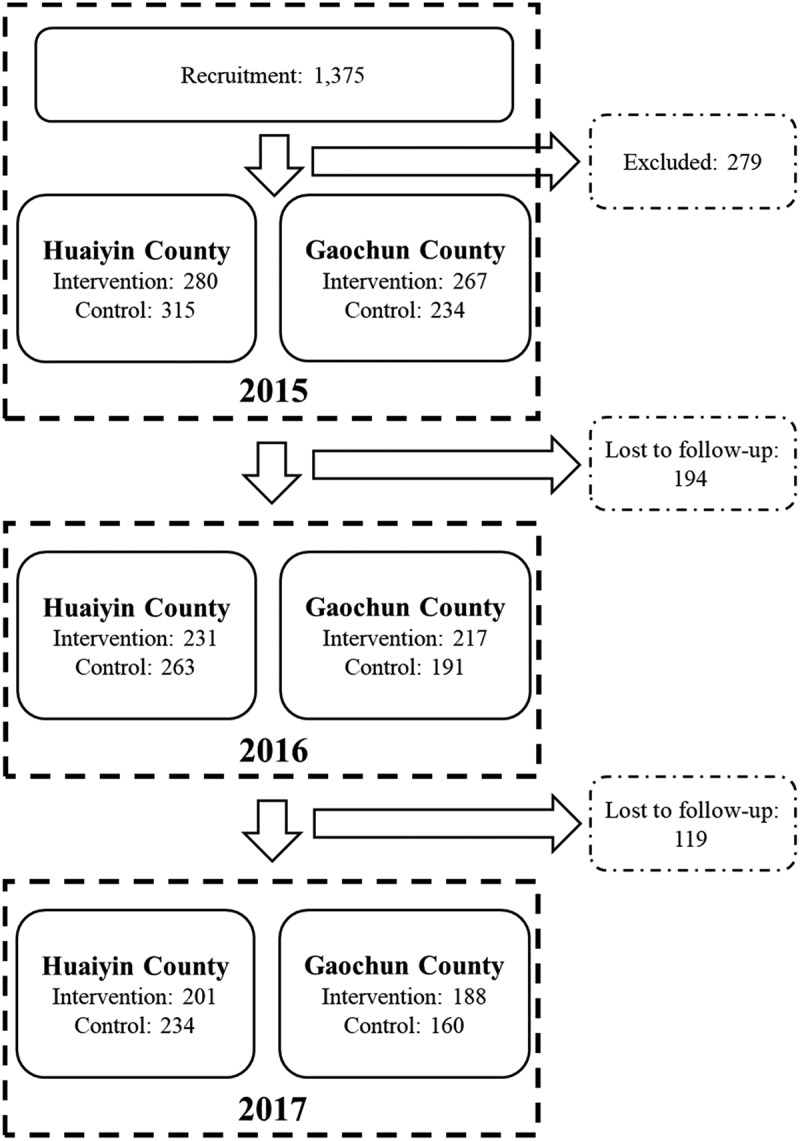
Recruitment of the patient participants

At baseline data collection in 2015, we recruited 1,096 participants according to the inclusion criteria. We lost to follow-up 194 and 119 participants in 2016 and 2017, respectively. Finally, we had 783 participants (435 in Huaiyin, 348 in Gaochun), of which 389 were in the intervention group, and 394 in the control group. The recruitment of patients is shown in Figure 1.

### Sociodemographic characteristics

Table 1 shows the sociodemographic characteristics of the participants at the baseline data collection. The mean age for all participants was 62.3 years, and the mean duration of T2DM was 7.3 years. More females participated in the study, with a proportion of 71.7% in the intervention group and 69.5% in the control group. Of the participants, 83.4% were married, and 76.9% had a lower educational level. The majority of the participants (87.2%) were farmers or doing housework. The proportion of participants in the intervention group, compared to the control group, was significantly higher in Gaochun than in Huayin county.

**Table 1.** Sociodemographic characteristics of participants at baseline data collection (2015)

	Intervention (389)			Control (394)		p
	n	%		n	%	
**Mean age (SD)**	62.4 (8.43)			62.3(8.20)		0.973
**Mean diagnosed year**^†^ **(SD)**	7.4 (5.05)			7.3 (5.16)		0.587
**County**						
Huaiyin	201	51.7		234	59.4	**0.030**
Gaochun	188	48.3		160	40.6	
**Sex**						
Male	110	28.3		120	30.5	0.503
Female	279	71.7		274	69.5	
**Marital Status**						
Single	62	15.9		68	17.3	0.620
Married	327	84.1		326	82.7	
**Educational level**						
Lower education	308	79.2		294	74.6	0.130
Higher education	81	20.8		100	25.4	
**Occupation type**						
Farming or house working	344	88.4		339	86.0	0.316
Other types of occupation	45	11.6		55	14.0	

†The diagnosed year means the years since being diagnosed with T2DM

### Mean difference in FBG level and diabetes knowledge score in the intervention and control group

**Figure 2.**
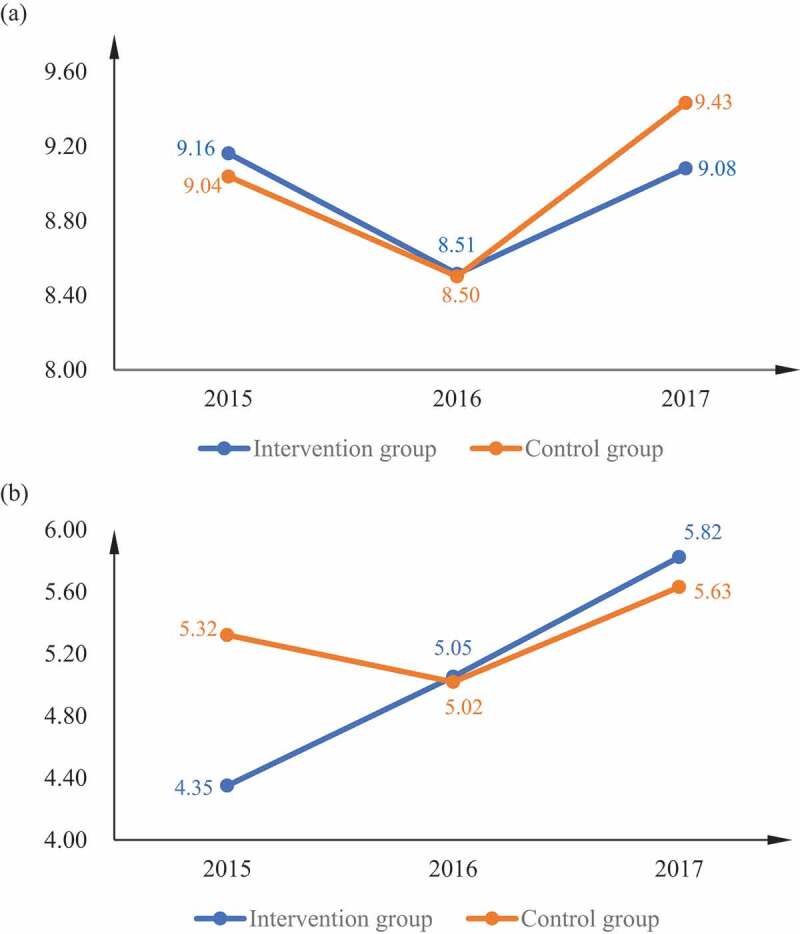
(a,b) The changes in FBG level (mmol/l, mean value) and knowledge score (mean value). (a) The changes of FBG level (mmol/l, mean value). (b) The changes of knowledge score (mean value)

Figure 2(a,b) illustrates the changes in diabetes knowledge score and FBG among participants in the intervention and control group, at baseline (2015) and two follow-ups (2016 and 2017) of data collection. The control group had a higher diabetes knowledge score than the intervention group in 2015. The intervention group experienced a continuous increase in the diabetes knowledge score in 2016 and in 2017. The diabetes knowledge score in the control group decreased in 2016, and then increased in 2017. Regarding the FBG level, in both the intervention and the control group, there was a decrease in 2016, and then an increase in 2017. In 2017, the FBG level was higher in the control group than in the intervention group.

Table 2 summarizes the comparison of diabetes knowledge score and FBG level between the intervention and control groups at baseline and two follow-ups. In 2016, the mean diabetes knowledge score in the intervention group increased significantly compared to 2015 (mean difference was 0.70, 95% CI 0.48, 0.93), while it decreased significantly in the control group (mean difference was −0.30, 95% CI −0.53, −0.82). Meanwhile, the FBG level decreased significantly in both the intervention and control group in 2016 compared with 2015, with the mean difference −0.65 (95% CI −0.90 to −0.39) and −0.54 (95% CI −0.81, −0.26), respectively. When comparing the diabetes knowledge score between 2016 and 2017, both the intervention and the control group increased significantly, while the intervention group increased more than the control group, with the mean difference being 0.77, 95% CI 0.53 to 1.01. The FBG level increased significantly in the two groups from 2016 to 2017, with the mean difference 0.45 (95% CI 0.11 to 0.80) in the intervention group, and 0.94 (95% CI 0.62 to 1.26) in the control group. When comparing the data in 2015 and 2017, no significant change of FBG level was found in either group. The mean diabetes knowledge score increased significantly by 1.47 (95% CI 1.24 to 1.71) and 0.31 (95% CI 0.09 to 0.53) in 2017 in the intervention and control group, respectively, compared with 2015.

**Table 2.** Comparison* of knowledge score (mean value) and FBG level (mmol/l, mean value) between the intervention group and control group, at baseline and two follow-ups

		**2015 vs. 2016**				**2016 vs. 2017**				**2015 vs. 2017**		
		Mean difference	95% CI	p		Mean difference	95% CI	p		Mean difference	95% CI	p
Knowledge score	Intervention	0.70	(0.48, 0.93)	**<0.001**		0.77	(0.53, 1.01)	**<0.001**		1.47	(1.24, 1.71)	**<0.001**
	Control	-0.30	(-0.53, -0.82)	**0.007**		0.61	(0.38, 0.84)	**<0.001**		0.31	(0.09, 0.53)	**0.006**
FBG	Intervention	-0.65	(-0.90, -0.39)	**<0.001**		0.45	(0.11, 0.80)	**0.009**		0.00	(-0.36, 0.36)	0.999
	Control	-0.54	(-0.81, -0.26)	**<0.001**		0.94	(0.62, 1.26)	**<0.001**		0.33	(-0.09, 0.75)	0.119

* Comparisons made using paired T-test

### Analysis using a mixed-effects linear regression model

0Table 3 shows the mixed-effects linear regression model for diabetes knowledge score and FBG level in both counties. Positive associations were found between diabetes knowledge score and the interaction of time and group, indicating that the diabetes knowledge score had improved significantly more among participants in the intervention group, compared to the control group. The beta coefficient was 0.96 (95% CI 0.69 to 1.24) in 2016 and 1.14 (95% CI 0.87 to 1.42) in 2017. Older participants had a lower score (beta coefficient −0.02, 95% CI −0.03 to −0.01) in 2017, while participants who had a longer duration of T2DM obtained a higher diabetes knowledge score (beta coefficient 0.02, 95% CI 0.01 to 0.04). Participants with higher educational level had a higher score than lower educated participants, the beta coefficient being 0.39 (95% CI 0.18 to 0.59).

**Table 3.** Mixed-effects linear regression model for diabetes knowledge score and FBG level and in both counties

	**Knowledge score**				**FBG**		
	Effect size*	p	95% CI		Effect size*	p	95% CI
**Time (Reference: 2015)**							
2016	-0.25	**0.012**	(-0.45, -0.06)		-0.57	**<0.001**	(-0.85, -0.28)
2017	0.33	**0.001**	(0.13, 0.53)		0.22	0.189	(-0.11, 0.55)
**Group (Reference: Control group)**							
Intervention	-0.97	**<0.001**	(-1.19, -0.75)		0.14	0.425	(-0.21, 0.49)
Time*Group (Reference: 2015*Control)							
2016*Intervention	0.96	**<0.001**	(0.69, 1.24)		-0.15	0.470	(-0.54, 0.25)
2017*Intervention	1.14	**0.007**	(0.87, 1.42)		-0.46	**0.042**	(-0.90, -0.02)
**Age**	-0.02	**<0.001**	(-0.03, -0.01)		-0.02	**0.028**	(-0.04, 0.00)
**Diagnose year**	0.02	**0.007**	(0.01, 0.04)		0.10	**<0.001**	(0.07, 0.12)
**Sex (Reference: male)**							
Female	-0.09	0.362	(-0.27, 0.10)		-0.02	0.878	(-0.34, 0.29)
**Educational level (Reference: lower education)**							
Higher education	0.39	**<0.001**	(0.18, 0.59)		-0.43	**<0.001**	(-0.78, -0.07)
**Occupation type (Reference: farming or house working)**							
Others	-0.08	0.532	(-0.32, 0.16)		-0.08	0.690	(-0.50, 0.33)
**Marital status (Reference: Single)**							
Married	0.02	0.876	(-0.20, 0.23)		-0.18	0.344	(-0.54, 0.19)
**(Constant)**	6.66	**<0.001**	(5.91, 7.40)		9.75	**<0.001**	(8.46, 11.02)

*Effect size is the beta coefficient

The overall FBG level declined significantly from 2015 to 2016, beta coefficient −0.57 (95% CI −0.86 to −0.28). The change from 2015 to 2017 was not statistically significant. However, the intervention had a positive impact on lowering participants’ FBG level in the intervention group in 2017, with a beta coefficient of −0.46 (95% CI −0.90 to −0.02). Older age or having a higher educational level were associated with a lower FBG level compared with others, the beta coefficient being −0.02 (95% CI −0.04 to 0.00) and −0.43 (95% CI −0.78 to −0.07), respectively. Participants with longer T2DM duration, however, had a significantly higher FBG level (beta coefficient 0.10, 95% CI 0.07 to 0.12) than those with shorter T2DM duration.

The mixed-effects linear regression models for diabetes knowledge score and FBG level were also done separately for Huaiyin and Gaochun county (Table 4). Similar to the overall analysis, the intervention positively affected the diabetes knowledge score in 2016 and 2017, in both Huaiyin and Gaochun county. The beta coefficient in Huaiyin was 0.69 (95% CI 0.28 to 1.09) in 2016, and 0.83 (95% CI 0.42 to 1.22) in 2017. The beta coefficient of the interaction between time and group on diabetes knowledge score in Gaochun was 1.23 (95% CI 0.88 to 1.59) in 2016 and 1.39 (95% CI 1.03 to 1.75) in 2017. A significant association between FBG level and the interaction of time and group, with the beta coefficient −0.76 (95% CI −1.32 to −0.19), suggesting a long-term effect of the intervention, was only found in Gaochun county in 2017. A stratified analysis by the patients’ sex (male vs. female), marital status (single vs. married), educational level (lower education vs. higher education), and occupation type (farming or house working vs. other types of occupation) showed similar results as the overall analysis (data not shown)

**Table 4.** Stratified mixed-effects linear regression models for diabetes knowledge score and FBG level in Huaiyin and Gaochun

**Huaiyin (North)**									**Gaochun (South)**						
	**Knowledge score**				**FBG**				**Knowledge score**				**FBG**		
	Effect size*	p	95% CI		Effect size*	p	95% CI		Effect size*	p	95% CI		Effect size*	p	95% CI
**Time (Reference: 2015)**															
2016	-0.33	**0.018**	(-0.61, -0.06)		-0.44	**0.022**	(-0.82, -0.06)		-0.14	0.298	(-0.41, 0.12)		-0.74	**<0.001**	(-1.15, -0.30)
2017	0.07	0.644	(-0.21, 0.34)		-0.10	0.698	(-0.62, 0.41)		0.72	**<0.001**	(-1.63, -1.04)		0.32	0.146	(-0.11, 0.75)
**Group (Reference: Control group)**															
Intervention	-0.64	**<0.001**	(-0.96, -0.32)		0.28	0.232	(-0.18, 0.73)		-1.34	**<0.001**	(-1.63, -1.04)		0.06	0.819	(-0.47, 0.60)
Time*Group															
2016*Intervention	0.69	**0.001**	(0.28, 1.09)		-0.50	0.068	(-1.04, 0.04)		1.23	**<0.001**	(0.88, 1.59)		0.24	0.411	(-0.33, 0.81)
2017*Intervention	0.83	**<0.001**	(0.42, 1.22)		0.40	0.279	(-0.32, 1.11)		1.39	**<0.001**	(1.03, 1.75)		-0.76	**<0.001**	(-1.32, -0.19)
**Age**	-0.02	**0.003**	(-0.03, -0.01)		-0.30	**0.004**	(-0.05, -0.01)		-0.03	**<0.001**	(-0.03, -0.15)		0.00	0.939	(-0.03, 0.03)
**Diagnose year**	0.03	**0.030**	(0.00, 0.05)		0.12	**<0.001**	(0.09, 0.16)		0.02	0.101	(-0.01, 0.04)		0.09	**<0.001**	(0.05, 0.12)
**Sex (Reference: male)**															
Female	-0.14	0.319	(-0.42, 0.14)		0.19	0.382	(-0.23, 0.61)		-0.01	0.922	(-0.25, 0.23)		-0.22	0.359	(-0.69, 0.25)
**Educational level (Reference: lower education)**															
Higher education	0.37	**0.009**	(0.09, 0.64)		-0.24	0.275	(-0.66, 0.19)		0.40	**0.019**	(0.07, 0.74)		-0.62	0.060	(-1.27, 0.03)
**Occupation type (Reference: farming or house working)**															
Others	-0.07	0.692	(-0.40, 0.27)		0.06	0.808	(-0.45, 0.58)		-0.01	0.969	(-0.35, 0.34)		-0.24	0.490	(-0.91, 0.44)
**Marital status (Reference: Single)**															
Married	-0.01	0.974	(-0.31, 0.30)		-0.31	0.190	(-0.78, 0.15)		0.04	0.812	(-0.25, 0.32)		0.05	0.864	(-0.51, 0.61)
**(Constant)**	6.54	**<0.001**	(5.52, 7.57)		10.09	**<0.001**	(8.51, 11.67)		7.01	**<0.001**	(5.90, 8.12)		8.70	**<0.001**	(6.56, 10.83)

*Effect size is the beta coefficient

## Discussion

The educational intervention improved the level of diabetes knowledge in the intervention group compared to the control group, at both first and second follow-up. Meanwhile, in the second follow-up (2017), the intervention had a significant impact on reducing FBG level in the intervention group, compared to the baseline. The stratified analysis suggested a greater impact of the intervention in Gaochun than in Huaiyin county. As the implementation of the intervention was in the hands of the local HFPC, the intervention may have been more strongly implemented in Gaochun than in Huaiyin county.

Participants in the intervention group from both Huaiyin and Gaochun county had improved diabetes knowledge level at the two-year follow-up, while participants in Gaochun improved more than participants in Huaiyin, with a higher mean knowledge score in both 2016 and 2017. In Huaiyin county, the FBG level did not differ significantly between the intervention and control group, in 2016 or in 2017. However, when comparing the FBG level in 2015 and 2017, participants in the intervention group from Gaochun county had a lower increase than in the control group. Although the content of the intervention was the same in both counties, the implementation was controlled by the local HFPC, and it is possible that the quality and intensity of implementation varied between the counties. The research team had no information regarding the implementation.

Some previous studies have reported the long-term (typically over one year) impact of educational interventions for patients with T2DM, in other settings [12–15]. The previous studies reported differences in the change in blood glucose control [12–14]. Johnson et al. conducted a two-year educational intervention for T2DM patients in USA [12], consisting of personal counseling by the study nurse, also customized to the patient [12]. The study reported that the blood glucose level, measured by HbA1c, for patients receiving the education was significantly lower than among patients not receiving education, after two years of observation [12]. Another study in England and Scotland showed no significant difference in the HbA1c level between patients with a structured group education programme, and patients with usual care, after a 3-year follow-up [13]. Meanwhile, Wing et al. compared an intensive lifestyle intervention (ILI) and diabetes support and education (DSE) for diabetes patients in a 4-year follow-up [14]. Participants with ILI had a better improvement in the reduction of blood glucose level, than participants with DSE [14]. Another study also reported an improvement in diabetes knowledge level for patients after having a 2-year PRECEDE (Predisposing, Reinforcing, Enabling, Causes in Educational Diagnosis, and Evaluation) education intervention [15]. We only found two Chinese language studies focused on China with an educational intervention longer than one year [16,17]. Both of the studies reported a significant decrease in the blood glucose level among patients with the intervention, while one of the studies found that the patients’ diabetes knowledge level also improved at 24-months follow-up [17]. It is difficult to quantitatively compare the impact of different educational intervention, because of the heterogeneity in design of the studies, education contents, evaluation methods, and disparity of assessment tools [26]. Although the impact on diabetes knowledge differed in different studies, we still believe it is necessary to conduct educational intervention among T2DM patients in rural China, as the diabetes knowledge level remains low. Our intervention had other components than only education (e.g. follow-up home visits, case management) which may also have contributed to the impact.

There are several limitations in the present study. The study was implemented in a real-life setting in PHC in rural China, which could be both a strength and a limitation. The implementation was done in a context where the research team did not have control over the implementation process. However, the intervention still obtained positive results, suggesting that such an intervention can successfully be conducted in rural China settings. Nevertheless, the research team did not assess adherence to the intervention or control details when performing the study. For example, we did not have information on why the diabetes knowledge score decreased in the control group in 2016. In addition, other policy changes may have interfered with the intervention studied. For example, Huaiyin and Gaochun county participated in a national government initiative project on chronic diseases held by National HFPC in late 2015 [26]. The routine health care services for patients in the control group may also have been performed differently in the different counties. The project aimed to encourage local HFPC to establish a comprehensive prevention and control demonstration zone for chronic disease. This may have contributed to the observed decrease in the FBG level in 2016 for participants both in the intervention and control group. When designing the content of the educational lecture and the questionnaire, simple and fundamental information on diabetes was emphasized. As a result, the impact on diabetes knowledge score was limited. However, the improvement of diabetes knowledge remained in the second year, while few other studies have shown changes maintained over time [27]. The proportion lost to follow-up was relatively high in the current study. One possible reason is the long duration of the intervention, which may increase risk of loss to follow-up [28]. On the other hand, there was no difference in socio-demographic characteristics between those who were lost to follow-up and those who stayed in the current study. Other factors in the bigger project, such as the new performance appraisals for health care professionals may also have had an influence on the results of the current study [18]. However, this could not be assessed in the present study. Selection bias might exist in the current study, as the participants were those who had a record in the Township health centres.

Our study used FBG as an outcome measure since the HbA1c test usually has a much higher cost, and because the HbA1c test could not be performed in some of the participating health centers. HbA1c may be a preferable measure as it reflects the blood glucose level over a longer time period [29]. However, studies comparing the accuracy and sensitivity of FBG and HbA1c for measuring blood glucose level have come to varied conclusions [29,30].

The present study shows that it might be important to do stratified analyses to assess if the impact of the intervention differs by geographic area, as the implementation of an intervention may vary, and the impact of other factors may also differ between areas. In our study, the implementation was controlled by local HFPC, which resulted in a lack of information on the fidelity of the implementation of the intervention. It is recommended to conduct an implementation assessment in cooperation with local HFPC.

The present study is part of a larger project which includes educational interventions for both T2DM patients and health care professionals from the PHC institutions in rural China [18]. Along with the previous three studies in the project, this study indicates that the diabetes care in rural China for patients can be improved at the PHC level, by increasing the collaboration between county-level hospitals and PHC and by providing education on T2DM and its management both to health care professionals in PHC and to patients with T2DM [19,20,23].

## Conclusion

The educational intervention improved the diabetes knowledge score in the intervention group, while no significant improvement was found in the control group in both year 2016 and 2017, suggesting that there may be a longer-term impact of an educational intervention. The intervention also had a positive impact on FBG level in the intervention group in 2017. Stratified analyses by county showed a differential impact, both regarding diabetes knowledge score and FBG level, suggesting that the local implementation of the intervention may have differed between counties. Combined with the findings regarding the influence of the intervention among health care professionals [23], the intervention had a positive influence both for patients and for health care professionals.

## Paper context

Educational interventions have been implemented in China to improve glucose control and diabetes knowledge for patients with type 2 diabetes mellitus, while knowledge is still lacking in rural China. The present study reported that the educational intervention improved the diabetes knowledge as well as the fasting blood glucose (FBG) level in rural China. Meanwhile, the educational intervention had a long-term (2 years) impact in improving the diabetes knowledge, and controlling the FBG level.

## Appendix A

**Table A1.** Key elements of the intervention

The actor	
Conducted by service team in each county.	
The service team consisted of:	One to two Diabetes experts from county-level hospitals (team leader)
	One public doctor from county-level CDC (team consultant)
	One nurse from county-level hospital
**The action**	
For patients in the intervention area:	1. Health education lectures
	1). Basic information on diabetes
	2). Self-management strategies
	3). Advice on physical exercise and diet therapy
	4). Advice to patients when having high blood glucose level
	5). Introduction of measuring blood glucose and severe acute complications
	6). Prevention of diabetes
	2. Periodical follow-up go-to-door visits & physical examination
	3. Special medical services
**Action target**	
For diabetes patient participants who live in the intervention area. Their FBG level was extracted from medical records, and their diabetes knowledge was tested by questionnaire.	
**Temporality**	
Huaiyin & Gaochun county: 2015–2017	
Jingjiang county: 2015–2016	
**Dose**	
Health education lecture: every two months.	
Periodical follow-up go-to-door visits: every two months; physical examination: once a year.	
Special medical services: performed when patient needed.	
**Implementation outcome affected**	
Diabetes knowledge: measured by nine questions from a questionnaire	
FBG level: extracted from medical records	

## Appendix B

**Table B1.** Questions related to diabetes knowledge

No.	Question	Options		
1.	Do you know the diagnose criteria of diabetes (FBG)? ^†^	Yes, it is ____ mmol/l	I do not know	
2.	Is dizziness a symptom of diabetes?	Yes^‡^	No	I do not know
3.	Is obesity a risk factor for diabetes?	Yes^‡^	No	I do not know
4.	Do people with a diabetes family history have higher risk of diabetes?	Yes^‡^	No	I do not know
5.	Is smoking or drinking a risk factor for diabetes?	Yes^‡^	No	I do not know
6.	Will monitoring your blood glucose help to control diabetes?	Yes^‡^	No	I do not know
7.	Will eating high fat or high sugar food help to control diabetes?	Yes	No^‡^	I do not know
8.	Will you be blind if the diabetes cannot be controlled?	Yes^‡^	No	I do not know
9.	Is it necessary to keep using medication if you have already controlled your blood glucose level?	Yes^‡^	No	I do not know

**^†^**The answer ‘Yes’, and FBG greater than 7 mmol/l, is classified as right answer.

^‡^The right answer.
